# Cytokine Induced Phenotypic and Epigenetic Signatures Are Key to Establishing Specific Macrophage Phenotypes

**DOI:** 10.1371/journal.pone.0078045

**Published:** 2013-10-21

**Authors:** Nicolai A. Kittan, Ronald M. Allen, Abhay Dhaliwal, Karen A. Cavassani, Matthew Schaller, Katherine A. Gallagher, William F. Carson, Sumanta Mukherjee, Jolanta Grembecka, Tomasz Cierpicki, Gabor Jarai, John Westwick, Steven L. Kunkel, Cory M. Hogaboam

**Affiliations:** 1 Department of Pathology, University of Michigan Medical School, Ann Arbor, Michigan, United States of America; 2 Division of Vascular Surgery, University of Michigan Hospital, Ann Arbor, Michigan, United States of America; 3 Novartis Institutes of Biomedical Research, Respiratory Disease Area, Horsham, West Sussex, United Kingdom; University Medical Center Freiburg, Germany

## Abstract

Macrophages (MΦ) play an essential role in innate immune responses and can either display a pro-inflammatory, classically activated phenotype (M1) or undergo an alternative activation program (M2) promoting immune regulation. M-CSF is used to differentiate monocytes into MΦ and IFN-γ or IL-4+IL-13 to further polarize these cells towards M1 or M2, respectively. Recently, differentiation using only GM-CSF or M-CSF has been described to induce a M1- or M2-like phenotype, respectively. In this study, we combined both approaches by differentiating human MΦ in GM-CSF or M-CSF followed by polarization with either IFN-γ or IL-4+IL-13. We describe the phenotypic differences between CD14^hi^ CD163^hi^ CD206^int^ FOLR2-expressing M-CSF MΦ and CD14^lo^ CD163^lo^ CD206^hi^ GM-CSF MΦ but show that both macrophage populations reacted similarly to further polarization with IFN-γ or IL-4+IL-13 with up- and down-regulation of common M1 and M2 marker genes. We also show that high expression of the mannose receptor (CD206), a marker of alternative activation, is a distinct feature of GM-CSF MΦ. Changes of the chromatin structure carried out by chromatin modification enzymes (CME) have been shown to regulate myeloid differentiation. We analyzed the expression patterns of CME during MΦ polarization and show that M1 up-regulate the histone methyltransferase MLL and demethylase KDM6B, while resting and M2 MΦ were characterized by DNA methyltransferases and histone deacetylases. We demonstrate that MLL regulates CXCL10 expression and that this effect could be abrogated using a MLL-Menin inhibitor. Taken together we describe the distinct phenotypic differences of GM-CSF or M-CSF MΦ and demonstrate that MΦ polarization is regulated by specific epigenetic mechanisms. In addition, we describe a novel role for MLL as marker for classical activation. Our findings provide new insights into MΦ polarization that could be helpful to distinguish MΦ activation states.

## Introduction

Macrophages (MΦ) are tissue resident cells of myeloid origin that are found in various organs where they contribute to immune responses by the killing of pathogens and the secretion of pro-inflammatory cytokines [1-3]. They also participate in the resolution of inflammation and have been described to promote wound healing [4]. These seemingly opposing functions have been ascribed to different polarization states of MΦ, the classically activated M1 phenotype and the alternatively activated or M2 phenotype, respectively [1-4]. 

IFN-γ alone or in combination with LPS is commonly used *in vitro* to induce a M1 phenotype, while IL-4 and IL-13 induce M2 MΦ. GM-CSF and M-CSF, growth factors traditionally used to differentiate monocytes into dendritic cells or MΦ, were recently found to alone promote a M1- and M2-like phenotype, respectively [5-8]. Thus, not only are specific cytokines critical for driving a M1 or M2 MΦ phenotype, but the polarization process is further complicated by the growth factors used for MΦ differentiation. 

Hematopoietic differentiation involves epigenetic modifications that are responsible for specific changes in the chromatin structure enabling or inhibiting the transcription of genes [9]. Using mouse models, our group and others have recently established a role for epigenetic modifications in post-septic DCs and in the alternative activation of macrophages [10-13]. However, less is known about the role of chromatin modifying enzymes (CME) in inflammation and the regulation of human innate immune responses [14,15]. 

In this current study we analyzed the expression of phenotypic markers in GM-CSF- and M-CSF-derived, IFN-γ- and IL-4+IL-13-polarized M1 and M2 MΦ. We define M-CSF MΦ as CD14^hi^ CD163^hi^ CD206^int^ cells that express FOLR2 and GM-CSF MΦ as CD14^lo^ CD163^lo^ CD206^hi^. In addition, we demonstrate that polarization with either IFN-γ or IL-4+IL-13 induces a distinct pattern of gene expression that is independent of the growth factors. Importantly, the mannose receptor CD206, a well-described marker for alternative activation [16], was highly expressed in all GM-CSF MΦ, even when compared to IL-4+IL-13-treated M-CSF MΦ.

Because of the importance of epigenetic changes during hematopoietic cell differentiation [9,17], we also looked at the expression of key CME during classical and alternative activation. M1 MΦ displayed elevated histone 3 lysine 4 trimethylation (H3K4me3) at the promoter site of the pro-inflammatory chemokine CXCL10 that correlated with an increased H3K4-specific methyltransferase mixed lineage leukemia (MLL) expression and activity. Notably, CXCL10 protein expression was decreased when M1 MΦ were pre-treated with a MLL inhibitor. M1 MΦ also showed an up-regulation of histone 3 lysine 27 (H3K27)-specific demethylase 6B (KDM6B), compared to the CME expression pattern in resting and M2 MΦ, which was characterized by DNA methyltransferases (DNMTs) and histone deacetylases (HDACs). Thus, our data suggests that MΦ polarization leads to distinct activation states in MΦ, which are associated with specific epigenetic patterns. In addition, we describe MLL as a novel marker for classical macrophage activation.

## Materials and Methods

### Monocyte isolation and MΦ culture

The study was conducted in accordance with the principles expressed in the Declaration of Helsinki and approved by the Institutional Review Board of the University of Michigan (HUM00001441). Blood samples were obtained from healthy donors after written informed consent. Peripheral blood mononuclear cells (PBMC) were obtained by a standard Ficoll gradient (GE Healthcare). Monocytes were isolated from PBMC using anti-CD14 magnetic beads (Miltenyi Biotec) and cultured at a concentration of 0.5-1.0 x 10^6^ cells/ml in complete RPMI 1640 medium (Lonza) containing 10% FBS (Cell Generation), 100 U/ml penicillin, 100 µg/ml streptomycin (Mediatech), supplemented with either 50 ng/ml M-CSF or GM-CSF (R&D Systems). On day+3 fresh media containing the growth factors was added. On day+7 the cells were washed twice with PBS containing Ca^2+^ and Mg^2+^ (Lonza) and fresh complete RPMI (for resting MΦ), complete RPMI containing 100 ng/ml IFN-γ (for M1) or 10 ng/ml IL-4 and IL-13 (for M2) (all cytokines were from R&D systems) was added for 24h-72h. In some experiments 40 μM of the MLL-Menin inhibitor MI-2-2 at a 1:500 dilution (kindly provided by J. Grembecka and T. Cierpicki) [18] or vehicle DMSO (Sigma-Aldrich) was added at day+6 and M1 polarization was carried out on day+7 in the presence of the inhibitor or DMSO as described.

### Isolation of mRNA, genomic DNA and quantification of total methylated DNA content

RNA and genomic DNA were isolated from adherent MΦ using the AllPrep DNA/RNA Mini Kit (Qiagen). In experiments with RNA isolation only, Trizol^®^ Reagent (Invitrogen) was used, followed by a column purification step with an additional on-column DNAse digestion (RNeasy Mini Kit, Qiagen). RNA and genomic DNA were quantified in a NanoDrop 1000 spectrometer (Thermo Scientific). Equal amounts of genomic DNA were analyzed for DNA methylation using the MethylFlash^TM^ and Imprint^®^ Methylated DNA Quantification Kits (Epigentek and Sigma-Aldrich) according to the manufacturers’ instructions. 

### Reverse transcription and quantitative real-time PCR

RNA was reverse-transcribed using random hexamer primers (Applied Biosystems) and M-MLV reverse transcriptase (Invitrogen) or iScript^TM^ cDNA Synthesis Kit (Bio-Rad). All primers were from AB Biosystems except for MRC1, which was custom made [19]. The following primers were bought from Applied Biosystems: ALOX15 (*Hs00609608_m1*), CDH1 (*Hs01013959_m1*), CCR7 (*Hs99999080_m1*), CXCL10 (*Hs00171042_m1*), DNMT1 (*Hs00154749_m1*), DNMT3A (*Hs01027166_m1*), FOLR2 (*Hs01044732_g1*), IL10 (*Hs00174086_m1*), IL12B (*Hs00233688_m1*), KDM6B (*Hs00389738_m1*), MLL (*Hs00610538_m1*), RPS6KA5 (*Hs00178054_m1*), and SMYD3 (*Hs00224208_m1*). Quantitative real-time PCR was performed with human glyceraldehyde-3-phosphate dehydrogenase (GAPDH) (Applied Biosystems) as housekeeping gene on a TaqMan 7500 sequence detection system (Applied Biosystems) and fold expression was normalized to resting MΦ using the ΔΔCt method. To compare the effect of growth factors, fold expression of GM-CSF MΦ was normalized to M-CSF MΦ. 

### PCR Array for the expression of chromatin modification enzymes (CME)

MΦ were differentiated in RPMI 5% FBS, 100 U/ml penicillin, 100 µg/ml streptomycin, 50 ng/ml M-CSF or GM-CSF for 7 days and polarized for 48h with either 100 ng/ml IFN-γ + 1 µg/ml LPS or 10 ng/ml IL-4 and IL-13. RNA was extracted and subjected to RNeasy MinElute cleanup (Qiagen). A total of 1 µg mRNA was reverse transcribed using the RT^2^ First Strand kit (SA Bioscience). Expression of CME was analyzed using RT^2^ Profiler^TM^ PCR Array PAHS-085 according to the manufacturer’s instructions and the web-based RT^2^ Profiler^TM^ PCR Array Data Analysis software (SA Biosciences). Because of genomic DNA contamination in one sample, Ct values ≤ 31 cycles were considered for analysis and normalized to the housekeeping genes beta-2-microglobulin (B2M), ribosomal protein L13a (RPL13A) and GAPDH.

### Flow cytometry of cell surface markers and intracellular staining

MΦ were harvested using Cellstripper^TM^ (Mediatech) and Fc receptors were blocked with human IgG (Sigma-Aldrich) for 30 min. Cells were stained for 25 min at 4°C. For intracellular staining, cells were fixed in 2% formaldehyde and then permeabilized using the Perm/Wash^TM^ buffer kit (BD Biosciences) followed by antibody incubation. The following antibodies were used: Biotinylated anti-CD14, FITC-conjugated anti-CD14, Pacific Blue^TM^-conjugated anti-CD64, FITC-conjugated anti-CD68, APC-conjugated anti-CD163, APC-conjugated anti-CD206, PE-conjugated anti-CXCL10 (Biolegend), PE-conjugated anti-CD206 (eBioscience), and Pacific Orange^TM^-conjugated streptavidin (Invitrogen). For the detection of histone marks rabbit anti-trimethylated H3K4 and anti-trimethylated H3K27 (Millipore) were used, followed by secondary stain with DyLight^TM^ 488-AffiniPure goat anti-rabbit IgG (Jackson ImmunoResearch). Flow cytometry data was collected on a BD Biosciences LSR II and analyzed using FlowJo software (Tree Star). 

### Preparation of nuclear extracts and histone 3 lysine 4 (H3K4)-methyltransferase activity assay

Nuclear extracts were prepared from MΦ 24h after polarization using EpiQuik^TM^ Nuclear Extraction Kit I (Epigentek) and stored at -80° C until analyzed. Protein content was quantified using Bradford assay (Bio-Rad) and the H3K4-specific methyltransferase activity was determined in equalized amounts of nuclear protein using the EpiQuik^TM^ Histone Methyltransferase Activity/Inhibition Assay Kit (H3-K4) (Epigentek) according to the manufacturer’s instructions. 

### Chromatin immunoprecipitation (ChIP) assay and quantitative real-time PCR

MΦ were polarized for 48h and ChIP was performed as described [13]. In brief, cross-linking was performed in a final concentration of 1% formaldehyde for 10 min at RT and cell pellets were stored at -80° C until analyzed. Cells were lysed for 10 min on ice in SDS lysis buffer supplemented with a protease inhibitor cocktail (Sigma Aldrich), syringe passed, and sonicated 3 x for 10 sec using a Branson Sonifier 450 (Branson Ultrasonics) to obtain DNA fragments ranging from 200 - 1000 bp length. Five percent of the total chromatin volume was put aside for the input control. The rest of the chromatin was then incubated with antibodies against trimethylated H3K4, trimethylated H3K27 or rabbit polyclonal IgG (Millipore) overnight at 4°C. Immune complexes were collected after incubation with a salmon sperm DNA/agarose A mix (Invitrogen) for 1h at 4°C. The pellet was washed and the chromatin was eluted 2 x for 15 min at room temperature with 5 min at 65°C at the end of the second elution. The combined eluates were reverse cross-linked for 5h at 65°C. Samples were stored overnight at -20°C, followed by a proteinase K digestion for 1h at 45°C. DNA was recovered by a phenol/chloroform/isoamyl alcohol extraction followed by an ethanol precipitation. Precipitated DNA was analyzed using quantitative real-time PCR on a TaqMan 7500 sequence detection system (Applied Biosystems) with primers specific for the promoter regions of CXCL10 [20], MRC1 [21], and ALOX15 [21].

### Immunofluorescence microscopy

Cytospin slides were fixed in 4% paraformaldehyde 4% sucrose for 20 min at RT. Cells were permeabilized with PBS 0.05% Tween 20 and Fc receptors were blocked with human FcR Blocking Reagent (Miltenyi Biotec) for 30 min at RT. The slides were stained with primary antibodies over night at 4°C followed by a secondary antibody stain for 1h at RT. The slides were mounted using ProLong^®^ Gold Antifade Reagent with DAPI (Invitrogen). The following antibodies were used: mouse anti-MLL_c_, rabbit anti-trimethylated H3K4 (Millipore), goat anti-rabbit Alexa Fluor^®^ 568, and anti-mouse Alexa Fluor^®^ 488 (Invitrogen). 

### Statistical analysis

Data were analyzed using GraphPad prism software Version 6 (GraphPad). Mann-Whitney U test and Kruskal-Wallis one-way ANOVA followed by Dunn’s multiple comparison were applied for statistical analysis and p-values ≤ 0.05 were considered significant.

## Results

### M-CSF and GM-CSF MΦ differ in morphology and surface marker expression

**Figure 1:**
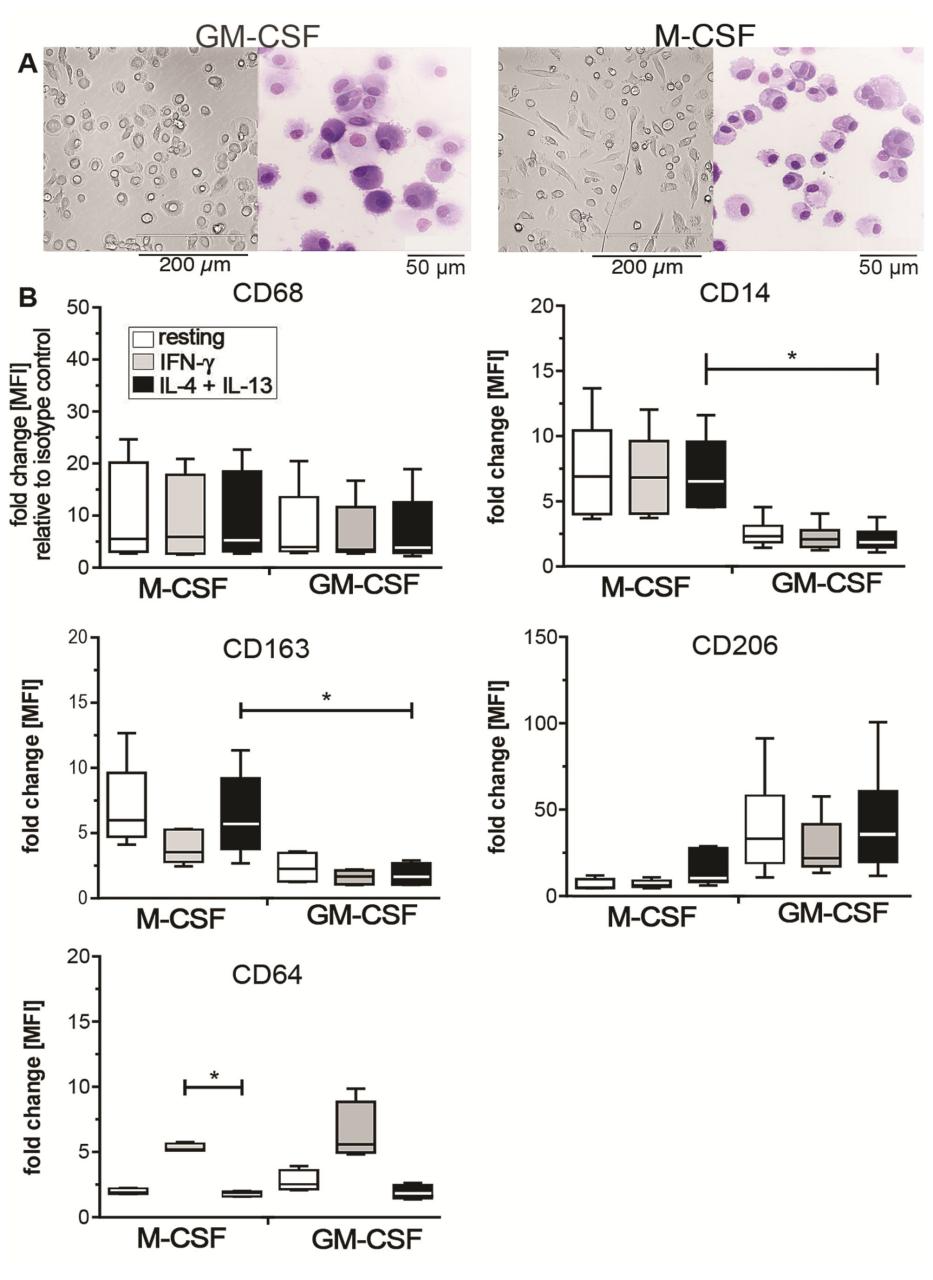
Phenotype of M-CSF and GM-CSF MΦ. (A) Cell culture images and cytospin slides of M-CSF and GM-CSF MΦ on day+7 of culture before polarization. Images of cell cultures and cytospins were taken at a 20X magnification with an EVOS microscope (AMG) and at a 40X magnification using a Olympus BX43 microscope and CellSens 1.7 Software (Olympus), respectively. Adobe Photoshop CS5.1 (Adobe Systems) was used to adjust brightness and contrast. (B) Flow cytometric measurement of surface marker expression depicted as mean fluorescence intensity (MFI) relative to the isotype control in box plots for M-CSF and GM-CSF MΦ (4-5 donors). White bars indicate resting MΦ, grey bars indicate IFN-γ stimulated M1 MΦ, and black bars indicate IL-4+IL13 stimulated M2 MΦ. Box plots represent the median and interquartile range, whiskers indicate the minimum and maximum, respectively. * p < 0.05.

Monocytes were differentiated into MΦ using either M-CSF or GM-CSF and subsequently polarized with IFN-γ (M1) or IL-4+IL-13 (M2). In our observations and as previously described [22], the majority of GM-CSF MΦ appeared to have a round-shaped morphology, while M-CSF MΦ cultures contained a higher number of spindle-like cells (Figure 1A). However, the extent of these features was donor-dependent. After withdrawal of the growth factors, we observed an increase in cell adherence upon IFN-γ, while resting and IL-4+IL-13-treated MΦ tended to detach from the plastic surface (data not shown). 

We next assessed the expression of MΦ markers CD68, CD14, mannose receptor CD206, the haptoglobin-hemoglobin scavenger receptor CD163, and the FcγRI CD64, 24h after polarization with IFN-γ or IL-4+IL-13 on M-CSF and GM-CSF MΦ by flow cytometry (Figure 1B). M-CSF and GM-CSF MΦ expressed similar levels of the intracellular MΦ marker CD68. While M-CSF MΦ retained the monocyte marker CD14 and highly expressed CD163, GM-CSF MΦ expressed the mannose receptor CD206 at higher levels than M-CSF MΦ. The mannose receptor is a known IL-4 inducible marker of alternative activation [16] and although we observed an increase of CD206 under M2 conditions in M-CSF treated cells, it did not reach the high expression levels seen in GM-CSF treated MΦ. Interestingly, the mannose receptor is also known to be highly expressed on human monocyte-derived dendritic cells (DC) commonly generated by culturing monocytes in the presence of GM-CSF and IL-4 [23]. But in contrast to DC that are considered to be a migratory, non-adherent cell population, the GM-CSF MΦ used in our experiments were strongly adherent. Apart from the increased levels of CD206 in M-CSF MΦ, IL-4+IL-13 did not significantly change the surface expression of the other markers tested. In contrast, IFN-γ led to an up-regulation of CD64 on both M-CSF and GM-CSF MΦ compared to resting MΦ while it down-regulated the expression of CD163 in M-CSF MΦ and of CD206 in GM-CSF MΦ (Figure 1B). Thus, in our experimental setting CD206 and CD163 were not definite markers for alternative activation in GM-CSF differentiated MΦ, as they were not regulated by IL-4+IL-13. In addition, CD163 is a marker of M-CSF differentiation rather than M2 polarization. In contrast CD64 is up-regulated by IFN-γ treatment in both M-CSF and GM-CSF differentiated MΦ and thus a valid marker for IFN-γ-induced classical MΦ activation. Notably, although we observed a significant down-regulation of CD14 on GM-CSF MΦ the cells were still able to respond to stimulation with LPS with the production of pro-inflammatory cytokines (data not shown).

### Polarized M-CSF and GM-CSF MΦ display a similar expression of M1 and M2 marker genes

**Figure 2:**
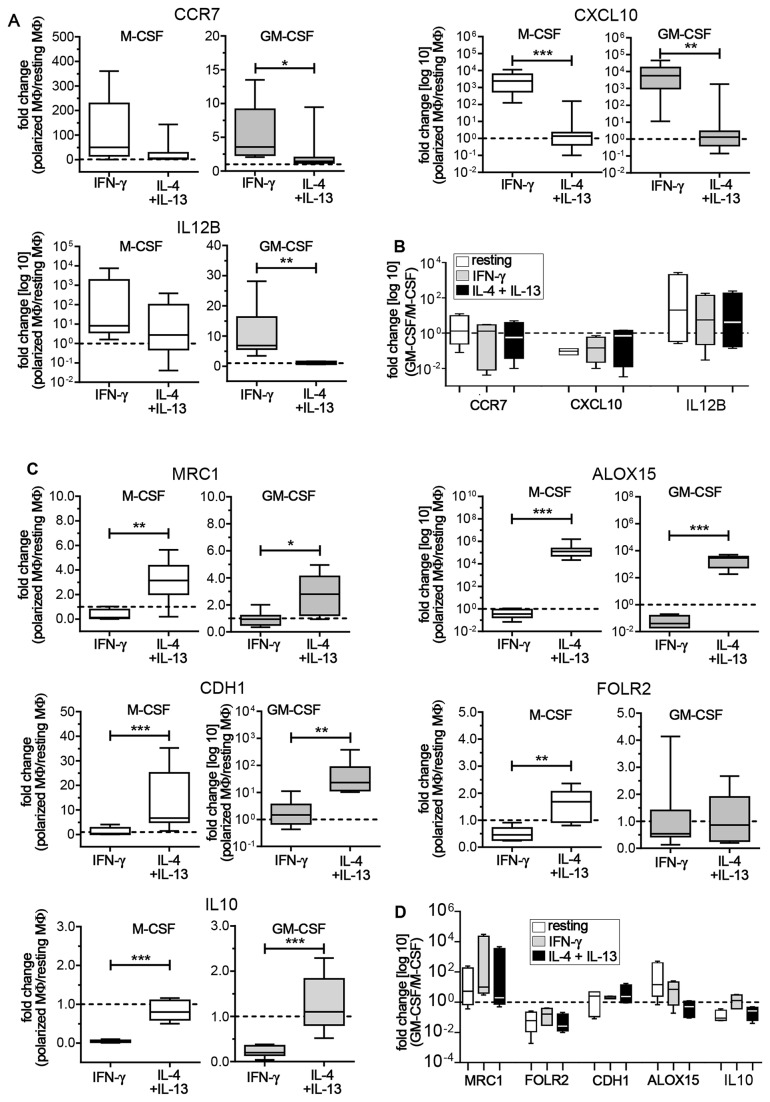
Expression of M1 and M2 marker genes. In all these figures resting MΦ expression levels are indicated by the dotted line. (A) M-CSF (white box plots) and GM-CSF MΦ (grey box plots) were analyzed for mRNA expression of M1 genes. Data are expressed as fold differences of polarized MΦ relative to resting MΦ (6-8 donors). (B) Fold expression of M1 genes in GM-CSF MΦ relative to M-CSF MΦ (4 donors). White bars indicate resting MΦ, grey bars indicate IFN-γ stimulated M1 MΦ, and black bars indicate IL-4+IL13 stimulated M2 MΦ. (C) M2 gene expression in polarized MΦ relative to resting MΦ (7-8 donors). (D) Fold expression of M2 genes in GM-CSF MΦ relative M-CSF MΦ (4 donors). Bars are as described above in (B). Box plots represent the median and interquartile range; whiskers indicate the minimum and maximum, respectively. * p < 0.05, ** p < 0.01, *** p < 0.001.

Resting, M1 and M2 MΦ were compared for expression of CCR7, CXCL10, and IL12B genes as representative markers for M1 and the mannose receptor (MRC1), e-cadherin (CDH1), folate receptor beta (FOLR2), 15-lipoxygenase (ALOX15) and IL10 genes as representative markers for M2 activation [7,19,21,24-26] (Figure 2). IFN-γ increased the expression of CCR7 and CXCL10 in both M-CSF and GM-CSF MΦ. While CXCL10 was significantly and similarly up-regulated in M-CSF and GM-CSF M1 as compared to M2 cells, the range and fold expression for CCR7 differed between growth factor treatments. IL12B was barely or not at all detected in resting MΦ. However, IFN-γ treatment led to a detectable increase in IL12B expression levels over M2 skewed cells in both M-CSF and GM-CSF MΦ (Figure 2A). The M2 markers MRC1, CDH1, ALOX15 and IL10 were all significantly up-regulated in IL-4+IL-13 treated cells regardless of the growth factor. In both M-CSF and GM-CSF MΦ ALOX15 mRNA was barely or not at all detectable in M1 MΦ, but was highly up-regulated upon IL-4+IL-13 treatment. In M-CSF MΦ, expression of FOLR2 showed a significant increase in M2 over M1 cells (Figure 2C).

We also compared the resting, M1 and M2 gene profiles of M-CSF and GM-CSF MΦ differentiated in parallel from a single donor (4 donors total) (Figure 2B+D). In line with previous reports [7,25], GM-CSF differentiated MΦ displayed a trend for higher levels of IL12B (Figure 2B) and lower levels of IL10 and FOLR2 (Figure 2D). Importantly, GM-CSF MΦ displayed elevated levels of MRC1 mRNA, thus confirming the increased mannose receptor expression that we observed in flow cytometry (Figure 2D). To a certain degree CDH1 and ALOX15, genes associated with a M2 phenotype [21,24,26], displayed a trend towards higher expression in GM-CSF over M-CSF treated MΦ. ALOX15 levels were highest in resting GM-CSF MΦ, somewhat higher in M1 MΦ after IFN-γ polarization and lower after IL-4+IL-13 stimulation. However, we observed considerable variation in expression levels among these genes in our human donors (Figure 2D). 

Overall, our results suggest that polarization with IFN-γ or IL-4+IL-13 leads to a similar gene expression pattern in both M-CSF and GM-CSF differentiated MΦ. In addition, GM-CSF MΦ displayed elevated levels of MRC1 mRNA, thus confirming the increased CD206 expression that we observed in flow cytometry.

### Different CME expression patterns of M1 and M2 MΦ

**Figure 3:**
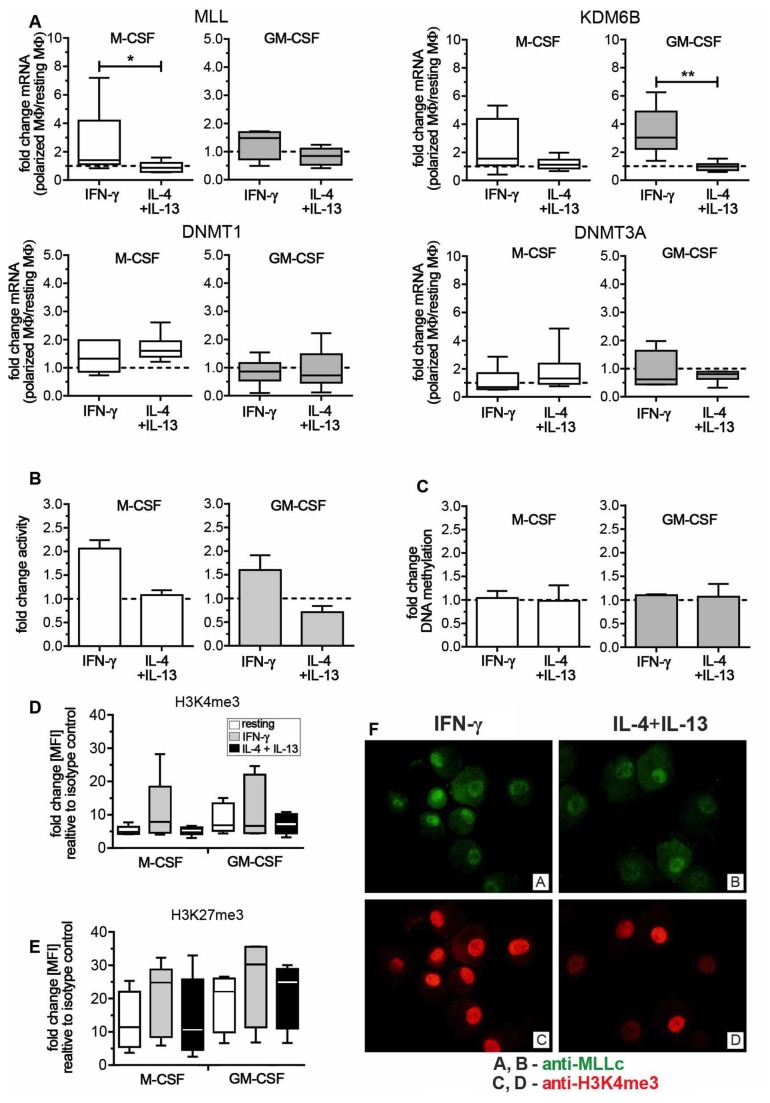
Expression and functional analysis of chromatin modifying enzymes (CME). In Figures A-C resting MΦ expression levels are indicated by the dotted line. (A) M-CSF (white box plots) and GM-CSF MΦ (grey box plots) were analyzed for mRNA expression of CME. Data are expressed as fold differences of polarized MΦ relative to resting MΦ (6-8 donors). Box plots represent the median and interquartile range, whiskers indicate the minimum and maximum, respectively. (B) Nuclear extracts of M-CSF (white bars) and GM-CSF MΦ (grey bars) were analyzed for H3K4-methyltransferase activity 24h after polarization (3 donors each). Data are expressed as relative absorbance of polarized MΦ relative to resting MΦ. Bars represent the median with range. (C) Genomic DNA of M-CSF (5 donors) and GM-CSF MΦ (4 donors) was analyzed for total DNA methylation 24h after polarization. Total DNA methylation is depicted as absorbance of polarized MΦ as relative to resting MΦ. Bars represent the median with range. (D) M-CSF MΦ and GM-CSF MΦ were polarized for 72h and stained for intracellular flow cytometry. MFI of H3K4me3 relative to the secondary antibody stain is shown for M-CSF and GM-CSF MΦ (5 donors). White bars indicate resting MΦ, grey bars indicate IFN-γ stimulated M1 MΦ, and black bars indicate IL-4+IL13 stimulated M2 MΦ. (E) MFI of H3K27me3 relative to the secondary antibody stain (4-5 donors). Bar codes are as described in (D). (F) Cytospin slides of M-CSF MΦ, polarized for 24h, were stained with anti-MLL_c_ (upper panel) and anti-H3K4me3 (lower panel). Slides were analyzed using an Olympus BX43 microscope (Olympus) with a X-Cite^®^120Q excitation light source (Lumen Dynamics) and CellSens1.7 Software (Olympus). Adobe Photoshop CS5.1 (Adobe Systems) was used to adjust brightness and contrast. * p < 0.05, ** p < 0.01.

Epigenetic changes constitute the underlying mechanism for hematopoietic cell differentiation [9] and distinct steps in human monocyte to MΦ differentiation are marked by changes in the epigenome [17]. In addition, our group and others have previously shown the involvement of epigenetic mechanisms during murine macrophage activation [10-12]. We therefore investigated whether this would also hold true for the activation of human MΦ in our model. First we used a PCR array to evaluate the role of CME during polarization of resting human MΦ to M1 and M2 MΦ. Under both M-CSF and GM-CSF conditions, M1 MΦ activation was associated with the up-regulation of MLL, KDM6B and the ribosomal protein S6 kinase (RPS6KA5) when compared to both resting and M2 MΦ (Table 1). In contrast, in both M-CSF and GM-CSF MΦ only few CME were up- or down-regulated in M2 MΦ when compared to resting MΦ. Interestingly, when compared to M1 MΦ, resting and M2 MΦ were both characterized by the preferential expression of DNA methyltransferases DNMT1, DNMT3A and B, and histone deacetylases (HDACs), all associated with gene silencing. For further analysis, we focused on the role of the activating CME MLL and KDM6B during classical activation and selected DNMT1 and DNMT3A as markers for resting/M2 MΦ. We next confirmed the expression of these CME via quantitative real-time PCR (Figure 3A). MLL and KDM6B were increased in M1 compared to M2 MΦ and this difference achieved significance for MLL in M-CSF differentiated and for KDM6B in GM-CSF differentiated MΦ. In contrast, DNMT1 and DNMT3A were similarly expressed in both M1 and M2 MΦ. 

**Table 1:** PCR Array for CME.

M-CSF MΦ				GM-CSF MΦ			
IFN-γ+LPS vs. resting				IFN-γ+LPS vs. resting			
*Gene*	*Fold up*	*Gene*	*Fold down*	*Gene*	*Fold up*	*Gene*	*Fold down*
KDM6B	3.41	AURKA	-2.69	AURKC	2.61	AURKB	-18.90
MLL	3.60	AURKB	-35.11	KDM6B	2.85	DNMT3A	-4.98
RPS6KA5	2.55	DNMT1	-4.65	MLL	3.03	DNMT3B	-5.81
		DNMT3A	-9.37	RPS6KA5	3.98	ESCO2	-23.31
		ESCO2	-63.86			HDAC2	-2.81
		HDAC2	-3.38			HDAC9	-3.46
		HDAC5	-4.52			KAT2A	-9.14
		HDAC9	-14.88			PRMT5	-124.62
		KAT2A	-19.87			SETD6	-3.08
		KAT6B	-3.80			SETD7	-3.09
		PRMT7	-3.33			SMYD3	-3.67
		SETD6	-3.14				
		SMYD3	-5.48				
IL-4+IL-13 vs. resting				IL-4+IL-13 vs. resting			
*Gene*	*Fold up*	*Gene*	*Fold down*	*Gene*	*Fold up*	*Gene*	*Fold down*
CIITA	2.84	AURKB	-9.82			ESCO2	-11.75
		ESCO2	-7.81			HDAC4	-3.85
		RPS6KA5	-3.08			NSD1	-2.80
IFN-γ+LPS vs. IL-4+IL-13				IFN-γ+LPS vs. IL-4+IL-13			
*Gene*	*Fold up*	*Gene*	*Fold down*	*Gene*	*Fold up*	*Gene*	*Fold down*
MLL	3.95	AURKB	-3.57	AURKA	2.59	AURKB	-8.53
RPS6KA5	7.86	DNMT1	-6.30	AURKC	3.34	DNMT1	-2.70
		DNMT3A	-9.66	CYDL	2.89	DNMT3A	-2.62
		ESCO2	-8.18	HDAC4	3.64	DNMT3B	-2.89
		HDAC2	-2.55	KDM6B	2.67	HDAC9	-8.07
		HDAC5	-3.05	KAT5	3.21	KAT2A	-5.41
		HDAC9	-18.05	KAT7	2.99	PRMT5	-157.68
		KAT2A	-18.98	RPS6KA5	8.72	SETD6	-2.87
		NEK6	-3.12	SETD1A	2.69		
		PRMT7	-3.88				
		SMYD3	-4.85				

Our results demonstrate that in general in both M-CSF and GM-CSF differentiated MΦ, polarization with IFN-γ leads to the up-regulation of MLL and KDM6B, while the CME pattern of IL-4+IL-13 treated MΦ does not significantly differ from resting MΦ.

### M1 MΦ display enhanced H3K4-methyltransferase activity and elevated H3K4me3

The histone demethylase KDM6B has been shown to regulate classical activation [27-29]. However, nothing is known about the role of MLL in MΦ polarization. MLL is a methyltransferase with a known specific activity for the lysine residue 4 of histone 3 (H3K4). Since trimethylation of H3K4 is a well-described marker of transcriptional activity [30] and we were able to show up-regulated MLL mRNA in M1 MΦ, we focused on the role of MLL during classical activation. We next analyzed whether we could also detect an increased histone methyltransferase enzyme activity during M1 polarization. Nuclear extracts from polarized M-CSF and GM-CSF MΦ were analyzed for H3K4-specific methyltransferase activity in a colorimetric assay (Figure 3B). In both M-CSF and GM-CSF MΦ, M1 displayed a higher activity when compared to resting or M2 MΦ. Conversely, the activity of M-CSF M2 was similar to resting MΦ, whereas GM-CSF M2 had a reduced activity. Together with the elevated mRNA levels this further suggested an increased MLL activity in M1 MΦ. 

In our PCR array, we initially found an up-regulation of DNMT1 and DNMT3A in M2 MΦ relative to M1 MΦ after 48h. But we did not observe significant changes in mRNA levels after 24h when we confirmed the array data using quantitative real-time PCR in 6 independent donors. However, this did not exclude a functional impact of DNMT enzymes on DNA methylation in M2 MΦ. In order to test for DNMT activity, we looked at changes in total DNA methylation. Genomic DNA from resting, M1 and M2 activated M-CSF and GM-CSF MΦ was isolated and tested for total DNA methylation (Figure 3C). In contrast to our initial findings from the PCR array, but corresponding to the data from our quantitative real-time PCR experiments, we did not observe major differences in overall DNA methylation. It is important to note that this approach aimed at detecting changes in the overall methylation of the genome. It does not exclude that both DNMTs mediate transcriptional silencing by increasing DNA methylation at discrete gene loci.

With elevated MLL levels and a higher H3K4-specific methyltransferase activity in M1 MΦ, we next looked at the mark set by the enzyme. We used flow cytometry to assess the methylation status of H3K4 72h after polarization (Figure 3D). We also looked at changes in H3K27 trimethylation, as this mark is a known target of the histone demethylase KDM6B, which was also up-regulated particularly in GM-CSF MΦ (Figure 3E) [27,28]. Despite some donor-dependent variability, M1 MΦ overall showed increased total H3K4 trimethylation (H3K4me3), when compared to either resting or M2 MΦ (Figure 3D). However and in contrast to the observed increase of KDM6B mRNA, we could not detect a decrease in total trimethylated H3K27 (H3K27me3). Instead H3K27me3 levels were also elevated in M1, when compared to resting or M2 MΦ, suggesting a potential role for repressive H3K27 methylation at later stages (i.e., 72 h) of MΦ polarization (Figure 3E). 

We next looked at MLL protein expression and activity by immunofluorescence (Figure 3F). Both M1 and M2 MΦ expressed MLL protein, however M1 M-CSF MΦ displayed an increase in H3K4me3 when compared to M2 MΦ. Overall, these findings are in agreement with the gene expression data as they confirm a higher degree of epigenetic regulation during classical activation, with a particularly important role for histone methylation at H3K4.

### Epigenetic remodeling of gene promoter sites in polarized M-CSF MΦ

**Figure 4:**
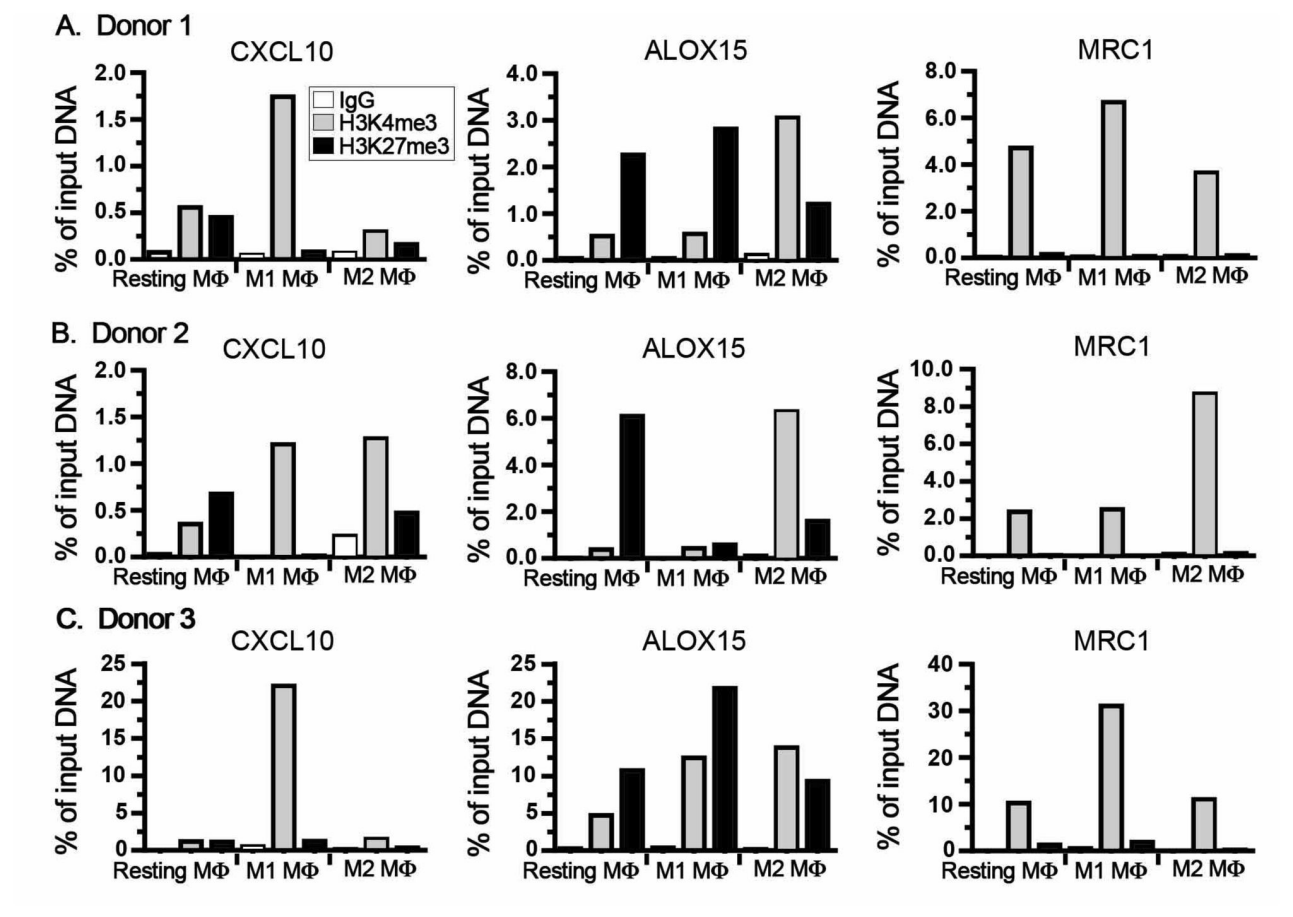
ChIP analysis for gene promoter-specific H3K4 and H3K27 trimethylation. For ChIP analysis M-CSF MΦ were polarized for 48h and the promoter regions of CXCL10, ALOX15 and MRC1 were analyzed for enrichment of H3K4me3 and H3K27me3. Rabbit polyclonal IgG was used as negative control. Data are expressed as percent of input DNA. Data from 3 independent donors for control IgG (white bars), H3K4me3 (grey bars) and H3K27me3 (black bars) are shown.

Our data implied that increased levels of total H3K4me3 accompany M1 activation in particular in M-CSF differentiated MΦ. To assess whether histone modifications also regulate MΦ polarization on a gene specific level in M1 and M2 MΦ, we analyzed the promoter regions of CXCL10, MRC1 and ALOX15 for changes in H3K4me3 and H3K27me3 using a ChIP approach (Figure 4). In line with our previous findings, the CXCL10 promoter region displayed an increase in H3K4me3 and simultaneous decrease in H3K27me3 in M1 when compared to resting or M2 MΦ, thus supporting our hypothesis that gene transcription at this site is regulated by histone modifications induced by MLL and KDM6B.

**Figure 5:**
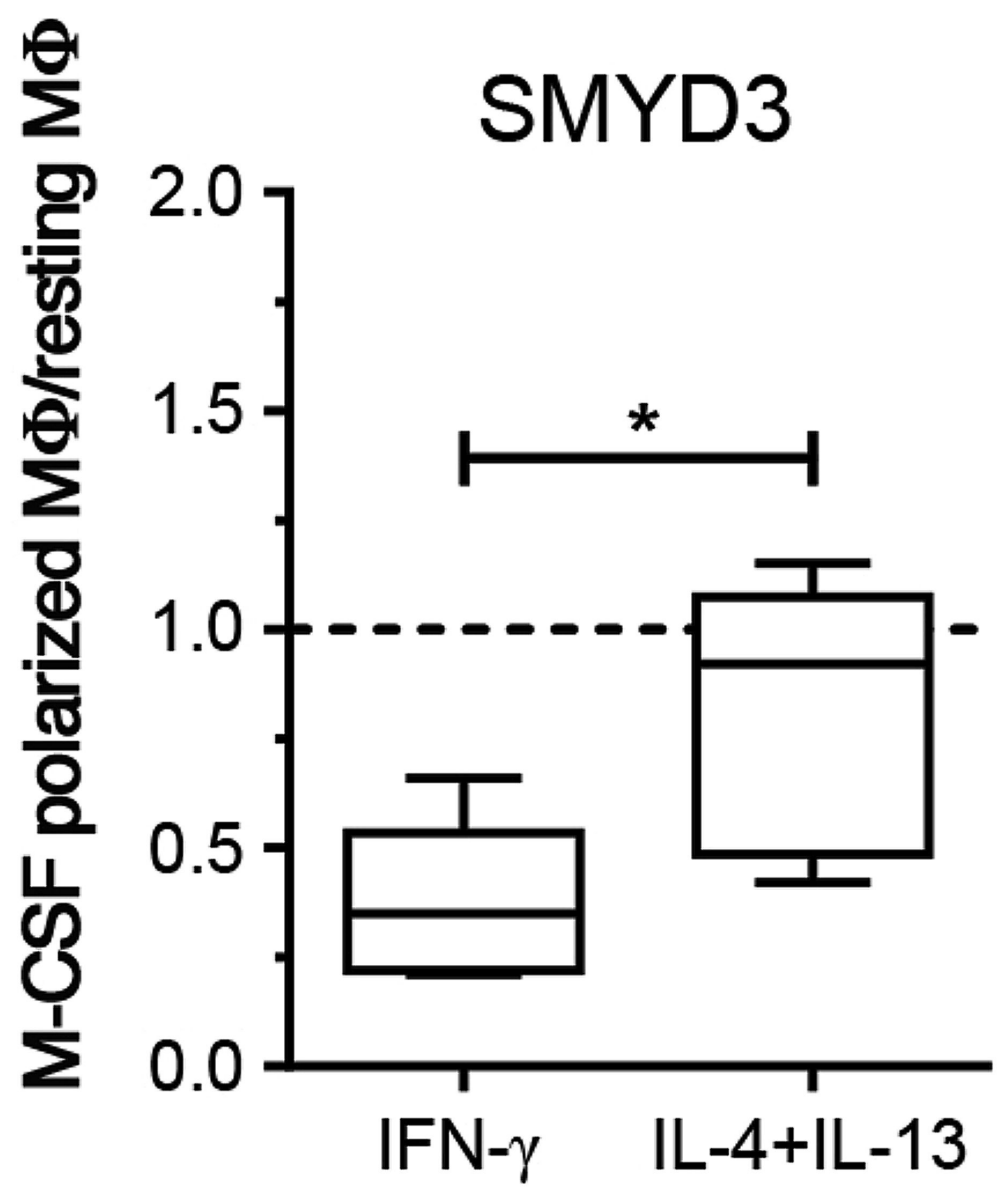
SMYD3 expression in M-CSF MΦ. M-CSF MΦ were polarized for 24h and analyzed for SMYD3 mRNA expression of M1. Data is expressed as fold differences of polarized MΦ relative to resting MΦ. Resting MΦ expression levels are indicated by the dotted line. (5 donors). Box plots represent the median and interquartile range; whiskers indicate the minimum and maximum, respectively. * p < 0.05.

Notably, the ALOX15 promoter in M2 was characterized by similar histone modifications with increased H3K4me3 and decreased H3K27me3. Thus, we re-evaluated our initial data from the PCR array (Table 1) focusing on H3K4-specific methyltransferases. SMYD3 was up-regulated in resting and M2 MΦ compared to M1 MΦ. We analyzed SMYD3 expression levels using quantitative real-time PCR and confirmed an increase of SMYD3 mRNA in M2 over M1 MΦ (Figure 5). It is important to note, that expression levels were decreased in M1- and M2-polarized MΦ when compared to resting M-CSF MΦ (dotted line in Figure 5).

Interestingly, the MRC1 promoter displayed a constitutively activated state in all MΦ indicated by increased H3K4me3 and low levels of H3K27me3 (Figure 4). This suggests that in contrast to CXCL10 and ALOX15, that seem to be regulated by polarization-specific CME (i.e. MLL and SMYD3), the expression of MRC1 and its gene product CD206 are regulated by other epigenetic or (post-) transcriptional mechanisms. 

These data further support our previous findings and demonstrate that epigenetic mechanisms regulate the expression of polarization-specific genes in M1 and M2 MΦ. In line with our hypothesis that MLL is involved during classical activation of MΦ, we were able to show that CXCL10 displays elevated H3K4me3 at its promoter site. 

### Targeting MLL-Menin interaction depletes CXCL10 protein expression in M1 MΦ

**Figure 6:**
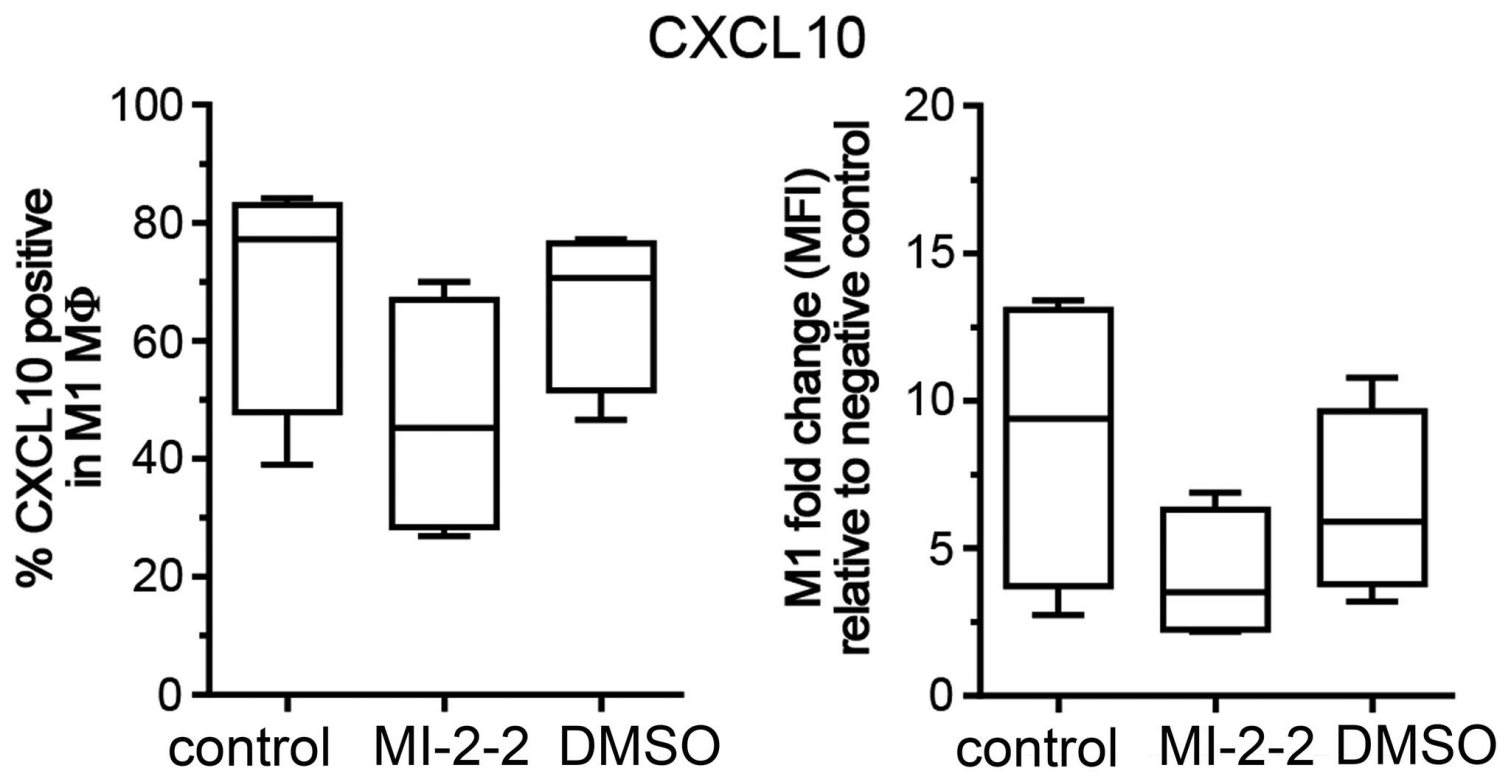
Effect of targeting MLL-Menin interaction on CXCL10 protein expression. Flow cytometric analysis of CXCL10 expression in M1 MΦ, pre-treated for 24h with 40µM of the MLL-Menin inhibitor MI-2-2 or DMSO 1:500. Results are shown as % of CXCL10 positive cells (left panel) and as MFI relative to the isotype control (right panel). Box plots represent the median and interquartile range, whiskers indicate the minimum and maximum, respectively.

To verify the role of MLL in epigenetically regulating CXCL10 expression and promoting M1 polarization, we next used a recently described inhibitor of the MLL-Menin interaction, MI-2-2, to specifically target MLL function [18]. Menin is a highly specific binding partner for MLL and has been shown to be a crucial co-factor for the leukemogenic activity of MLL-fusion proteins [31]. MΦ were treated with 40 μM of MI-2-2 or vehicle DMSO 24h prior to polarization with IFN-γ and CXCL10 expression was assessed using flow cytometry (Figure 6). MI-2-2 decreased the number of CXCL10 positive cells compared to untreated M1 MΦ and DMSO control (median 45.3%, range 26.9-70% vs. median 77.15%, range 39-84.1% vs. median 70.65%, range 46.6-77.2%, respectively). These findings demonstrate that inhibiting MLL function using a small molecule approach successfully interferes with CXCL10 production and thus with M1 function. 

## Discussion

**Figure 7:**
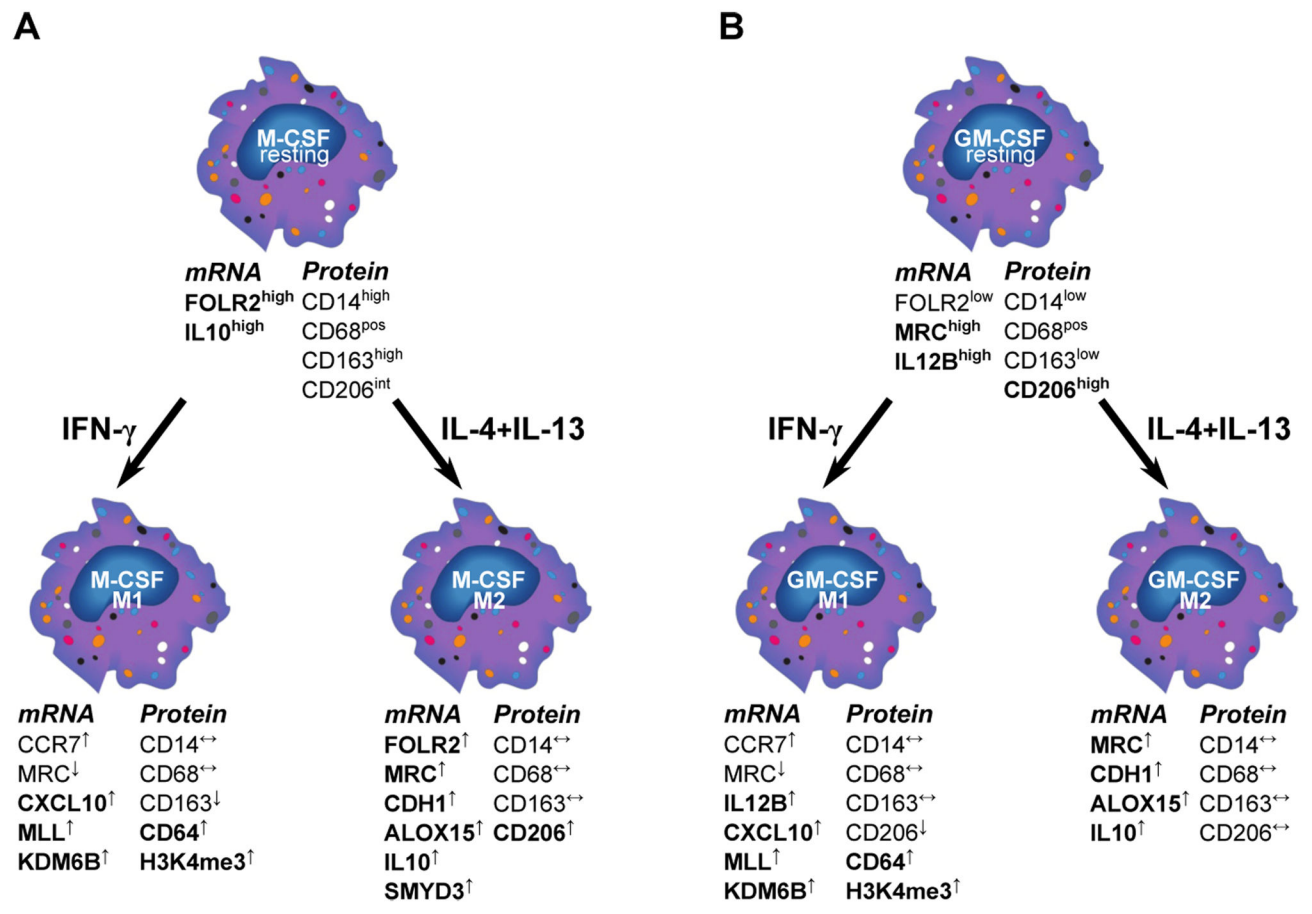
Phenotype of polarized M-CSF and GM-CSF MΦ. MΦ that were differentiated in M-CSF (A) and GM-CSF (B) were further polarized using IFN-γ or IL-4+IL-13. Characteristic features of M-CSF vs. GM-CSF MΦ are depicted in the upper part. Additional changes found in this study, induced by the polarization process are shown in the lower section with characteristic genes, surface markers and epigenetic changes for M-CSF and GM-CSF M1and M2 MΦ.

Polarized MΦ are found during infections and tissue repair, where their role is fundamental in shaping the immune response towards inflammation or tolerance [1-4]. Circulating monocytes differentiate into tissue MΦ upon exposure to growth factors [1]. In-vitro, this behavior is mimicked by plastic adherence of monocytes in the presence of M-CSF. When added to M-CSF MΦ, IFN-γ±LPS is commonly used to generate M1 whereas IL-4±IL-13 generates M2 MΦ [2,3]. More recently, this view has been challenged by the finding that GM-CSF alone is able to induce M1-like MΦ, characterized by an IL-12^hi^ IL-10^lo^ phenotype, while M-CSF differentiation leads to IL12^lo^ IL-10^hi^ MΦ corresponding to an M2-like cell type [5-8]. Thus, most studies investigating classical or alternative activation focused either on M1/M2 polarized M-CSF MΦ [21,24] or pointed out differences between M-CSF and GM-CSF MΦ [5-8,25]. It is conceivable however, that *in vivo*, monocytes simultaneously encounter growth factors and T cell-derived IFN-γ and/or IL-4+IL-13 depending on the prevailing tissue microenvironment. In order to better understand MΦ polarization, we combined growth factor treatments with cytokine-induced activation. In line with previous reports [25,32-34] we demonstrated that, independent of cytokine treatment, M-CSF MΦ display a CD14^hi^ CD163^hi^ CD206^int^ surface phenotype and express FOLR2. Conversely, GM-CSF MΦ were CD14^low^ CD163^low^ CD206^hi^ with elevated IL-12B expression (Figure 7). IFN-γ led to an up-regulation of CXCL10 and CD64 in both GM-CSF and M-CSF MΦ, whereas IL-4+IL-13 induced MRC1, ALOX15, CDH1, and FOLR2 mRNA expression (Figure 7). Notably, FOLR2 seemed to be associated with M-CSF MΦ rather than GM-CSF MΦ, which is in agreement with a previous report [25]. The mannose receptor CD206 was up-regulated upon IL-4+IL-13 in M-CSF MΦ, and showed even higher expression levels in all GM-CSF MΦ. Also there was a higher level of the mannose receptor gene MRC1 in resting and polarized GM-CSF MΦ. This is remarkable as CD206 is a well-described M2 marker and associated with an IL-4-driven pathophysiology [16,35], whereas GM-CSF has been described in the context of inflammation [6]. In fact, IL-4 treated M-CSF MΦ clearly up-regulated CD206, although it never reached the level of expression observed in GM-CSF MΦ. GM-CSF and IL-4 are used for the generation of dendritic cells (DC) and a high expression of the mannose receptor and the loss of CD14 surface expression are also common features of monocyte-derived DC [23,36]. It is interesting to speculate whether GM-CSF induces a DC-like phenotype in MΦ. Both DC and GM-CSF MΦ are major producers of IL-12 and our data indicate that GM-CSF not only induces CD206 expression but also down-regulates CD14 on the cell surface. These findings suggest that markers that are up-regulated during GM-CSF differentiation of MΦ seem to overlap with markers found in DC. However, the MΦ analyzed in this study were adherent while DC are considered to be a migratory, loosely or non-adherent cell population. Notably, a critical role for GM-CSF has been proposed in various (auto) inflammatory diseases [37].

Although the phenotypic changes described in this manuscript are derived from *in vitro* experiments in which MΦ were differentiated under controlled conditions by adherence to a plastic surface, the markers described herein have been demonstrated to exert *in vivo* relevance in human diseases. CD163 and CD206 expression on tissue MΦ has been used to distinguish an alternative activation state from classical activation in many human diseases like asthma, obesity and atherosclerosis [38-40]. Interestingly, to some extent these studies have raised the question about the validity of specific markers to characterize a distinct phenotype as well as about the “true nature” of MΦ polarization in human diseases. For example, adipose tissue MΦ, although displaying a M2-like phenotype with expression of CD206 and CD163, have been shown to secrete considerable amounts of pro-inflammatory cytokines [40]. Conversely, lung MΦ in the bronchoalveolar lavage of patients with asthma, a disease generally associated with a M2 MΦ phenotype, expressed less CD163 than the MΦ from healthy non-asthmatics [38]. Taken together our data support these and other previous *in vitro* findings [32-34] and suggest that the mannose receptor (CD206) or CD163 as single defining markers of alternative activation have to be re-evaluated. For the characterization of polarized MΦ in human diseases it is thus necessary to consider the effects of growth factors as well as cytokines and include a more comprehensive panel of phenotypic markers, e.g. CD14, CD206, CD163, FOLR2 and IL12B in the analysis.

But irrespective of their differences with regard to morphology, gene and surface receptor expression, M-CSF and GM-CSF MΦ appear to respond to IFN-γ or IL-4+IL-13 in a similar fashion with up- and down-regulation of common M1 and M2 genes (Figure 7), indicating that the transcription program initiated by these cytokines is independent of the growth factors and suggests that polarization-specific transcription factors control the expression of these markers [41].

Embryonic development and cellular differentiation are accompanied by considerable changes in the chromatin structure including DNA methylation and histone modifications. These modifications regulate gene transcription and silencing and are carried out by distinct CME. Notably, hematopoietic development and monocyte to MΦ differentiation are governed by changes in the epigenome [9,17]. Thus, it is conceivable that epigenetic mechanisms also control MΦ polarization [14,15]. Our group and others have previously demonstrated the involvement of epigenetic mechanisms in the alternative activation of murine macrophages [10-12] and in post-septic DC [13]. However, much less is known about epigenetics during human MΦ polarization. In this study we investigated if modulations in CME expression and function accompany human MΦ polarization and thus could serve as additional markers to better understand and define the M1 and M2 phenotype. We demonstrated that in M-CSF and GM-CSF MΦ, M1 up-regulated the activating CME MLL and KDM6B. Conversely, resting and M2 MΦ were characterized by CME associated with transcriptional repression including members of the DNMT and HDAC families. As H3K4me3 is a well-described marker of active gene expression [30], we further investigated the role of MLL in the process of classical activation. We demonstrated increased H3K4-specific methyltransferase activity in M1 that was accompanied by an increase of total H3K4me3 in flow cytometry. In addition, M1 MΦ displayed an enrichment of H3K4me3 in the promoter region of the M1 marker gene CXCL10. We demonstrated the functional relevance of MLL-mediated M1 gene expression, as we were able to deplete CXCL10 protein expression using a previously described MLL inhibitor that disrupts the binding of MLL to Menin [18,31]. Notably, MLL has been shown to regulate NF-κB-dependent gene expression after LPS and TNFα treatment [42] and NF-κB is an important activator of inflammatory gene transcription that regulates IFN-γ-induced CXCL10 transcription [43,44]. Thus, our experiments support these findings and underscore the role of MLL during M1 polarization. Notably, some of the changes observed upon IFN-γ stimulation were more pronounced in M-CSF than in GM-CSF MΦ, either suggesting that GM-CSF MΦ are already activated in a M1-like mode [5-8] or that other/additional CME play a role during classical activation of these cells. 

The role of KDM6B during MΦ polarization remains controversial. In contrast to murine MΦ, in which KDM6B mediates alternative activation [11,12], we found an up-regulation of KDM6B in classically activated MΦ. This is in agreement with previous reports showing an increase of KDM6B after LPS treatment [27,28]. We observed a decrease of H3K27me3 levels at the CXCL10 promoter during classical activation, but M1 MΦ did not display a reduction of total H3K27me3. However, treatment of human MΦ with a selective KDM6B inhibitor has been shown to reduce TNF production after LPS challenge [29].

Unexpectedly, alternative activation did not reveal a specific set of up-regulated CME in either M-CSF or GM-CSF MΦ, when compared to resting MΦ. When compared to classically activated MΦ, the M2 pattern of regulation resembled the one observed when M1 were compared to resting MΦ with preferential expression of DNMTs and HDACs. Selecting DNMT1 and DNMT3A as markers for M2, we found comparable expression levels and a similar amount of methylated DNA in M1 and M2 MΦ. However, at the promoter site of ALOX15 we also found an increase of H3K4me3 upon M2 polarization, indicative of methyltransferase activity. We hypothesize that this is due to SMYD3 activity, as we found that SMYD3, another H3K4-methyltransferase, is preferentially expressed in M2 MΦ. Recently, SMYD3 has been shown to regulate 15-Lipoxygenase expression [45] which further supports the data from our experiments. However, M2 clearly displayed a lower methyltransferase activity than M1 MΦ, suggesting that a high H3K4-specific methyltransferase activity is indeed a feature of classical activation (Figure 7).

Notably, the MRC1 promoter region displayed elevated H3K4me3 and low levels of H3K27me3 independent of polarization conditions. This indicates that MRC1 transcription is not regulated by polarization-induced changes in histone methylation. Interestingly, MRC1 expression also seems to be independent of c-MYC transcription factor binding that was shown to regulate M2 gene expression [21]. Considering its induction by IL-4 and GM-CSF, this suggests that mannose receptor expression in MΦ is regulated by other, yet unknown mechanisms.

In conclusion, we describe the phenotypic differences between CD14^hi^ CD163^hi^ CD206^int^ FOLR2-expressing M-CSF and CD14^lo^ CD163^lo^ CD206^hi^ GM-CSF MΦ. We also show that M-CSF and GM-CSF MΦ reacted similarly upon activation with IFN-γ or IL-4+IL-13 with the induction of well described M1 and M2 marker genes, respectively. This indicates that the transcription program initiated by IFN-γ or IL-4+IL-13 is independent of the growth factors, suggesting that transcription factors might control the expression of marker genes [41]. Furthermore and to the best of our knowledge we have demonstrated for the first time, the importance of histone modifications, in particular methyltransferase activity, during classical activation and introduce the H3K4-methyltransferase MLL as a potential novel marker for M1 polarization. 
